# Alteration of the Early Development Environment by Maternal Diet and the Occurrence of Autistic-like Phenotypes in Rat Offspring

**DOI:** 10.3390/ijms22189662

**Published:** 2021-09-07

**Authors:** Kinga Gawlińska, Dawid Gawliński, Ewelina Kowal-Wiśniewska, Małgorzata Jarmuż-Szymczak, Małgorzata Filip

**Affiliations:** 1 Department of Drug Addiction Pharmacology, Maj Institute of Pharmacology Polish Academy of Sciences, Smętna Street 12, 31-343 Kraków, Poland; gawlin@if-pan.krakow.pl (D.G.); mal.fil@if-pan.krakow.pl (M.F.); 2 Institute of Human Genetics, Polish Academy of Sciences, Strzeszyńska 32, 60-479 Poznań, Poland; ewelina.kowal@igcz.poznan.pl (E.K.-W.); malgorzata.jarmuz-szymczak@igcz.poznan.pl (M.J.-S.)

**Keywords:** autism spectrum disorder, ASD, epigenetics, high-fat diet, HFD, maternal diet, prefrontal cortex, pregnancy and lactation, offspring behaviors

## Abstract

Epidemiological and preclinical studies suggest that maternal obesity increases the risk of autism spectrum disorder (ASD) in offspring. Here, we assessed the effects of exposure to modified maternal diets limited to pregnancy and lactation on brain development and behavior in rat offspring of both sexes. Among the studied diets, a maternal high-fat diet (HFD) disturbed the expression of ASD-related genes (*Cacna1d*, *Nlgn3*, and *Shank1*) and proteins (SHANK1 and TAOK2) in the prefrontal cortex of male offspring during adolescence. In addition, a maternal high-fat diet induced epigenetic changes by increasing cortical global DNA methylation and the expression of miR-423 and miR-494. As well as the molecular changes, behavioral studies have shown male-specific disturbances in social interaction and an increase in repetitive behavior during adolescence. Most of the observed changes disappeared in adulthood. In conclusion, we demonstrated the contribution of a maternal HFD to the predisposition to an ASD-like phenotype in male adolescent offspring, while a protective effect occurred in females.

## 1. Introduction

The importance of nutrition to neonatal health during fetal development is highlighted by decades of studies with the Developmental Origins of Health and Disease (DOHaD) theory, which proposes that adverse in-utero conditions can influence developmental pathways in early life that result in long-term changes to offspring health and disease susceptibility [1,2]. Most of the current literature focuses on evaluating the influence of the composition of nutrients in the maternal diet on peripheral tissues and metabolic diseases. Despite the key role of maternal nutrition in the development and function of the offspring brain [3,4], a limited number of studies leave many interesting questions unanswered. It has been shown that maternal obesity and exposure to a high-fat diet (HFD) during early development are associated with an increased risk of developing serious mental health and behavioral disorders, including anxiety, depression, attention deficit hyperactivity disorder (ADHD), and schizophrenia, in offspring [5]. Recent preclinical and clinical findings also suggest a strong relationship between maternal overweight or obesity and the risk for autism spectrum disorder (ASD) in offspring (children born to obese mothers have a 36% higher risk of developing ASD) [6,7,8,9]. Moreover, the type and amount of fatty acids consumed by the mother before and during pregnancy could significantly influence the risk of having a child with ASD [10]. ASD is a highly heritable and heterogeneous neurodevelopmental disorder characterized by significant social, communication, emotional, and behavioral challenges, often not allowing those with ASD to lead a normal life in society and to have a reduced quality of life [11]. In the 21st century, the number of people diagnosed with ASD is still growing, and the sex ratio is three to one (boys to girls) [12]. Therefore, the search for the pathogenic mechanisms and risk factors for this complex disease, as well as understanding the sex differences that lead to a significantly more frequent diagnosis of ASD in boys, is very important.

Maternal obesity, often induced by consumption of an HFD at a key stage in the offspring’s life, could be responsible for epigenetic modifications, including changes in DNA methylation mechanisms responsible for fetal programming [13]. Methylation of gene promoters and regulatory regions impedes the binding of transcription factors, which leads to altered gene expression [14]. DNA methylation reprogramming is dynamic during embryonic development and the early postpartum period, which corresponds to the peak time of synaptogenesis. The maternal diet is a source of methyl donors, while DNA methylation is established in the developing fetus and may directly affect the epigenome of the offspring [15]. Abnormal DNA methylation observed in ASD patients could be caused by mutations in the genes involved in the epigenetic machinery or locus-specific changes localized in regulatory regions of genes [16].

A clear association between the maternal diet during pregnancy and lactation and an increased risk of ASD symptom development in offspring has not yet been established. We sought to address this knowledge gap by assessing the impact of maternal modified diets in the early stages of fetal brain development on adolescent and young adult offspring behavior.

Bearing in mind the above information, this study aims to identify the influence of a maternal HFD, high-carbohydrate diet (rich in sucrose; HCD), and mixed diet (rich in fat and carbohydrate; MD) limited to pregnancy and lactation on the expression profile of ASD-related genes within the offspring prefrontal cortex (PFCx)—one of the brain structures related to the pathogenesis of ASD symptoms [17]. Next, in male and female offspring, we assessed, in a longitudinal study (from adolescence to early adulthood), the impact of the maternal HFD on both the changes in social and repetitive behaviors and the changes in ASD-related proteins and epigenetic mechanisms related to DNA methylation or microRNA (miRNA) expression.

## 2. Results

### 2.1. A Maternal Modified Diet Disrupts the Expression of ASD-Related Genes in the Offspring PFCx

**Figure 1:**
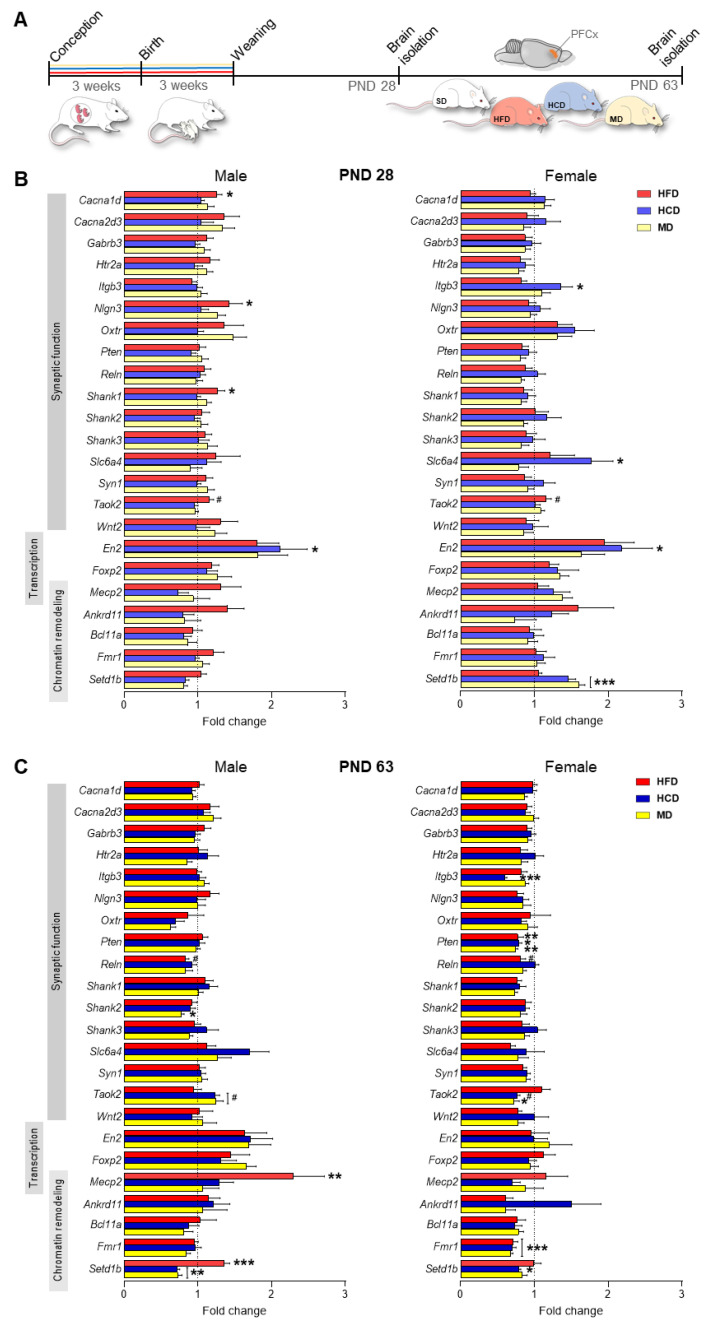
Maternally modified diets during pregnancy and lactation change the expression of genes associated with autism spectrum disorder (ASD) in the prefrontal cortex (PFCx) of adolescent and adult offspring. (**A**) Schematic of the experimental design. Rat dams were fed a standard diet (SD) or modified diets (high-fat diet (HFD), high-carbohydrate diet (HCD), and mixed diet (MD)) during pregnancy and lactation. After weaning, offspring were changed to the SD and sacrificed at postnatal day (PND) 28 and PND 63 to evaluate the changes in the expression of selected genes associated with ASD in the PFCx. (**B**,**C**) Effects of the modified maternal diets on the expression of ASD-related genes (compared to the SD groups; relative expression vs. SD) in the PFCx of male and female offspring at PND 28 (adolescence, B) and PND 63 (adulthood, C). Detailed results of statistical analysis are presented in Table 1. Data are expressed as the mean ± SEM. *n* = 10 male and 10 female rats/experimental group. Data were analyzed by two-way ANOVA and the Bonferroni multiple comparison post hoc test. ^#^ *p* < 0.07, * *p* < 0.05, ** *p* < 0.01, *** *p* < 0.001 versus SD.

To investigate the effect of maternal modified diets (HFD, HCD, and MD) consumed during pregnancy and lactation on proper brain development in offspring, we analyzed the expression of 23 ASD-related genes responsible for proper synaptic function, chromatin remodeling, and transcription in the PFCx (Figure 1, Table 1). In adolescent male offspring (PND 28), maternal HFD significantly increased the expression of the *Cacna1d* (*p* < 0.05), *Nlgn3* (*p* < 0.05), and *Shank1* (*p* < 0.05) genes, and a similar trend was observed for *Taok2* (*p* = 0.07); maternal HCD elevated *En2* expression (*p* < 0.05) compared to the SD group (Figure 1B). In females, compared to the control group (SD), there was an increase in the expression of *Itgb3* (*p* < 0.05), *Slc6a4* (*p* < 0.05), *En2* (*p* < 0.05), and *Setd1b* (*p* < 0.001) in the HCD group; *Setd1b* in the MD group (*p* < 0.001); and *Taok2* in the HFD group (*p* = 0.07) (Figure 1B).

In adult offspring (PND 63), we observed a different pattern of changes in the expression of the studied genes compared to the SD group (Figure 1C). In males, a maternal diet rich in fat significantly increased the expression of *Mecp2* (*p* < 0.01) and *Setd1b* (*p* < 0.001), with a simultaneous tendency to decrease the mRNA level of *Reln* (*p* = 0.07). In turn, maternal exposure to an HCD induced a decrease in *Setd1b* expression (*p* < 0.01), and a similar effect was observed in male MD offspring (*p* < 0.01). Additionally, the maternal MD reduced *Shank2* levels (*p* < 0.05). In the case of females, all studied diets reduced the expression of *Pten* (*p* < 0.05) and *Fmr1* (*p* < 0.001) in adulthood. The maternal HCD additionally decreased the levels of *Itgb3* (*p* < 0.001) and *Setd1b* (*p* < 0.05), while the maternal MD decreased *Taok2* expression (*p* < 0.05).

### 2.2. Maternal HFD during Pregnancy and Lactation Promotes Autistic-like Behaviors in Adolescent Male Offspring

**Figure 2:**
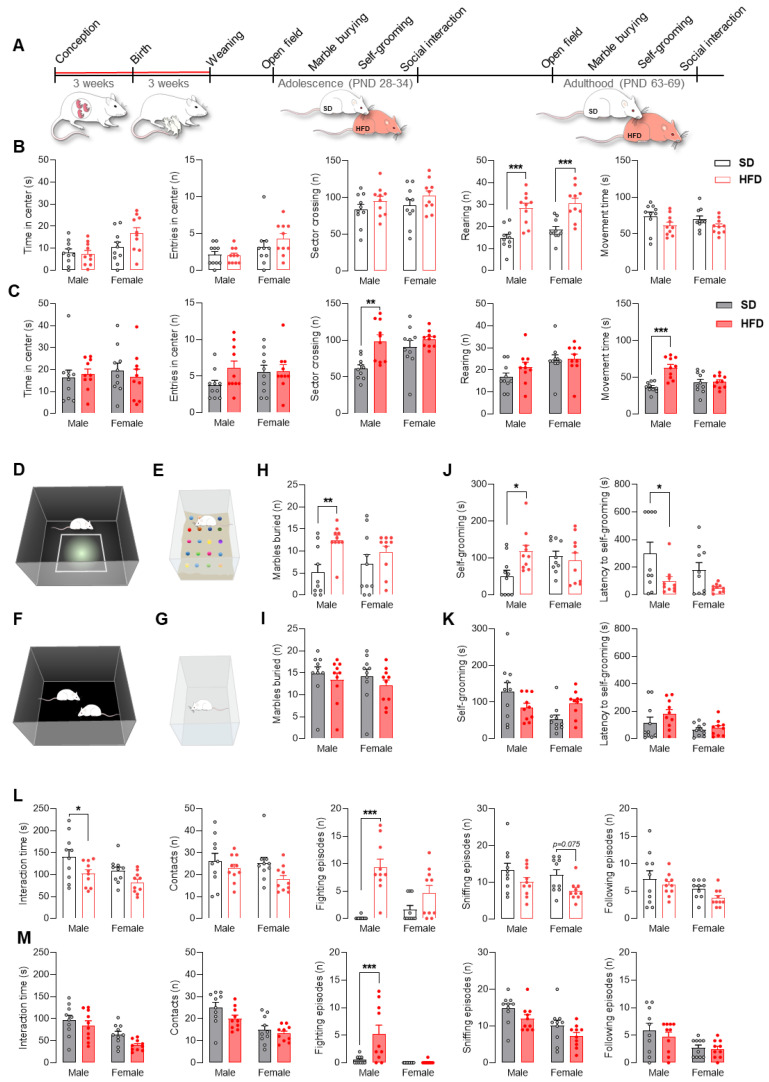
A maternal high-fat diet (HFD) during pregnancy and lactation provokes autism-like behavior in adolescent male offspring. (**A**) Schematic of the behavioral test timeline. Rat dams were fed a standard diet (SD) or HFD during pregnancy and lactation. After weaning, offspring were changed to the SD and tested in adolescence and adulthood. (**B**,**C**) Effect of the maternal HFD on the time spent in the center, number of entries into the center, sector crossings, and rearings, and the total movement time of male and female offspring in the open field test at postnatal day (PND) 28 (B, empty bars) and PND 63 (C, filled bars). PND 28: Rearing (main effect of diet: F(1, 36) = 41.11, *p* < 0.001), diet × sex interaction: F(1, 36) = 0.12, *p* = 0.73). PND 63: Sector crossing (main effect of diet: F(1, 36) = 11.66, *p* < 0.01), diet × sex interaction: F(1, 36) = 3.50, *p* = 0.07); Movement time (main effect of diet: F(1, 36) = 14.50, *p* < 0.001), diet × sex interaction: F(1, 36) = 12.56, *p* < 0.01). (**D**) Schematic of the open field test. (**E**) Schematic of the marble burying test. (**F**) Schematic of the social interaction test. (**G**) Schematic of the self-grooming test. (**H**,**I**) Effect of the maternal HFD on the number of marbles buried by male and female offspring at PND 30 (H, empty bars) and PND 65 (I, filled bars). PND 28: MB (main effect of diet: F(1, 36) = 9.03, *p* < 0.01), diet × sex interaction: F(1, 36) = 1.91, *p* = 0.17). (**J**,**K**) Effect of the maternal HFD on the time self-grooming and the latency to self-grooming of male and female offspring at PND 32 (J, empty bars) and PND 67 (K, filled bars). PND 28: SG (main effect of diet: F(1, 36) = 2.67, *p* = 0.11), diet × sex interaction: F(1, 36) = 5.19, *p* < 0.05); Latency (main effect of diet: F(1, 36) = 9.75, *p* < 0.01), diet × sex interaction: F(1, 36) = 0.45, *p* = 0.51). (**L**,**M**) Effect of the maternal HFD on the social interaction time, number of contacts, fighting episodes, sniffing episodes, and following episodes of male and female offspring in the social interaction test at PND 34 (L, empty bars) and PND 69 (M, filled bars). PND 28: Interaction time (main effect of diet: F(1, 36) = 8.69, *p* < 0.01), diet × sex interaction: F(1, 36) = 0.26, *p* = 0.62); fighting episodes (main effect of diet: F(1, 36) = 30.60, *p* < 0.001), diet × sex interaction: F(1, 36) = 7.90, *p* < 0.01); sniffing episodes (main effect of diet: F(1, 36) = 7.12, *p* < 0.05), diet × sex interaction: F(1, 36) = 0.15, *p* = 0.70). PND 63: Fighting episodes (main effect of diet: F(1, 36) = 8.40, *p* < 0.01), diet × sex interaction: F(1, 36) = 7.71, *p* < 0.01). Data are expressed as the mean ± SEM. *n* = 10 male and 10 female rats/experimental group. Data were analyzed by two-way ANOVA and the Bonferroni multiple comparison post hoc test. * *p* < 0.05, ** *p* < 0.01, *** *p* < 0.001 versus SD.

Given that ASD is a neurodevelopmental disorder that manifests early in life and often affects boys more than girls [18], for further research, we chose the HFD because it exhibited the greatest changes in the expression of the studied genes in male offspring 28 days after birth (Figure 1B). In four behavioral tests used in preclinical ASD studies, we assessed the behavior of adolescent and adult offspring exposed to a maternal HFD (Figure 2A,D–G).

In the open field test, adolescent offspring (males and females) exposed to maternal HFD showed a significantly greater number of rearing events (*p* < 0.001) (Figure 2B). Increased sector crossing (*p* < 0.01) and movement time (*p* < 0.001) in the open field area were observed in adult males exposed to a maternal HFD (Figure 2C).

To assess repetitive behavior in animals prenatally exposed to a maternal HFD, we used two tests: the marble burying test and the self-grooming test. The results indicated that only adolescent males from the HFD group buried more than twice as many marbles as control animals (*p* < 0.01) (Figure 2H). Moreover, at PND 32, males exposed to an HFD spent more time on self-grooming (*p* < 0.05) (Figure 2J), while exhibiting decreased the latency time before the first grooming episode during the test (*p* < 0.05) (Figure 2J). The changes observed in adolescence did not persist into adulthood (Figure 2I,K).

Rodents are very social animals and intrinsically exhibit a high level of reciprocal social interactions, communal nesting, parenting behaviors, and territorial scent marking. We assessed the behavior of the animals during the reciprocal social interaction test. Adolescent male (but not female) offspring exposed to a maternal HFD showed a reduced interaction time with unfamiliar rats (*p* < 0.05). At the same time, no differences in the number of contacts were recorded, which indicates a shortening of individual interaction episodes between animals. Additionally, in males from the HFD group, at PND 34, a significantly increased number of fighting episodes (*p* < 0.001) was observed (Figure 2L). Adolescent females exposed to a maternal HFD showed a tendency toward a reduced number of sniffing episodes (*p* = 0.075). In contrast to adolescent rats, adult rats showed no change in the length of social interactions. On the other hand, males from the HFD group displayed more frequent episodes of fighting (*p* < 0.001) (Figure 2M).

### 2.3. Maternal HFD Alters Global DNA Methylation and the DNA Methylating Enzymatic Machinery in Male Adolescent Offspring PFCx

**Figure 3:**
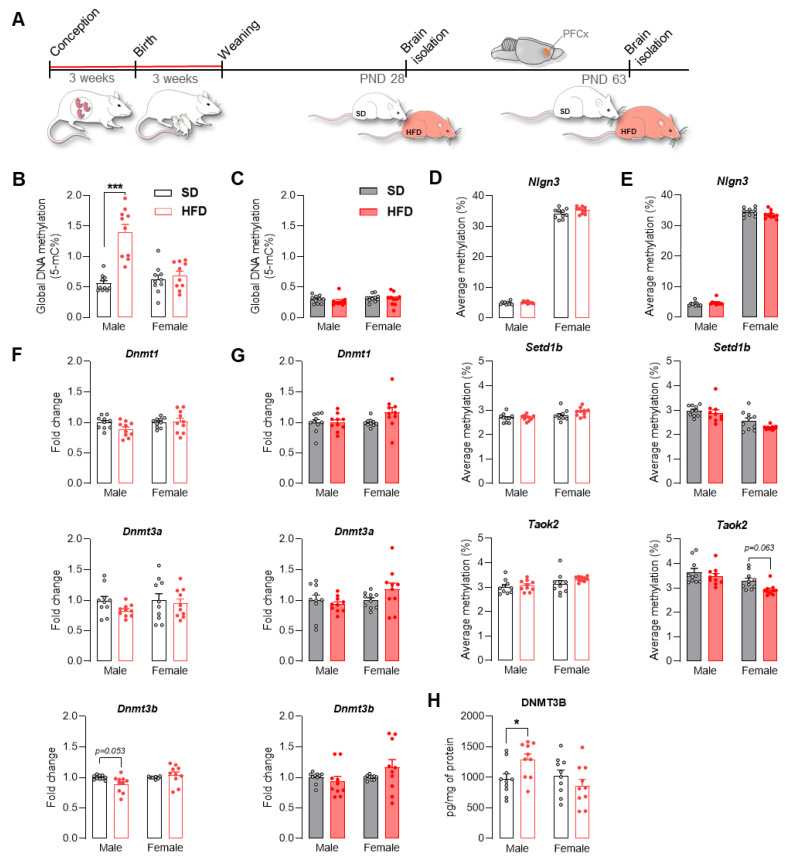
A maternal high-fat diet (HFD) during pregnancy and lactation increased global DNA methylation in the prefrontal cortex (PFCx) of adolescent male offspring. (**A**) Schematic of the epigenetic analysis. Rat dams were fed a standard diet (SD) or HFD during pregnancy and lactation. After weaning, offspring were changed to the SD and sacrificed at postnatal day (PND) 28 and PND 63 to evaluate epigenetic modifications in the PFCx. (**B**,**C**) Effect of the maternal HFD on global DNA methylation in the PFCx of male and female offspring at PND 28 (B, empty bars) and PND 63 (C, filled bars). PND 28: GM (main effect of diet: F(1, 36) = 28.98, *p* < 0.001), diet × sex interaction: F(1, 36) = 20.92, *p* < 0.001). (**D**,**E**) Effect of the maternal HFD on the promoter CpG island methylation of Nlgn3, Setd1b, and *Taok2* in the PFCx of male and female offspring at PND 28 (D, empty bars) and PND 63 (E, filled bars). PND 63: *Taok2* (main effect of diet: F(1, 36) = 5.18, *p* < 0.05), diet × sex interaction: F(1, 36) = 0.78, *p* = 0.38). (**F**,**G**) Effect of the maternal HFD on the Dnmt1, Dnmt3a and Dnmt3b mRNA levels in the PFCx of male and female offspring at PND 28 (F, empty bars) and PND 63 (G, filled bars). PND 28: Dnmt3b (main effect of diet: F(1, 36) = 1.02, *p* = 0.32), diet × sex interaction: F(1, 36) = 5.11, *p* < 0.05). (**H**) Effect of the maternal HFD on DNMT3B protein levels in the PFCx of male and female offspring at PND 28. DNMT3B (main effect of diet: F(1, 36) = 0.73, *p* = 0.40), diet × sex interaction: F(1, 36) = 6.60, *p* < 0.05). Data are expressed as the mean ± SEM. *n* = 10 male and 10 female rats/experimental group. Data were analyzed by two-way ANOVA and the Bonferroni multiple comparison post hoc test. * *p* < 0.05, *** *p* < 0.001 versus SD.

The maternal diet can influence the proper functioning of the brain and other tissues in the offspring by influencing epigenetic mechanisms, including DNA methylation, which can result in altered gene expression [19]. Looking for a potential mechanism that could be responsible for the altered expression of ASD-related genes in offspring exposed to an HFD, we assessed the level of 5-mC DNA methylation in the PFCx (Figure 3A) and showed that only in male offspring was the global DNA methylation level changed significantly and that it was higher than in male offspring from the control group (*p* < 0.001) (Figure 3B). No changes were observed in global DNA methylation in adult offspring regardless of sex (Figure 3C). The maternal HFD did not influence the expression of *Dnmt1*, *Dnmt3a*, and *Dnmt3b* in juvenile and adult offspring (Figure 3F,G), with the exception of a trend toward decreased expression of *Dnmt3b* in males at PND 28 from the maternal HFD group (*p* = 0.053). On the other hand, an increase in the protein level of DNMT3B was observed in adolescent offspring from the HFD group (*p* < 0.05) (Figure 3H). Despite the changes in the global DNA methylation level, detailed analysis of the DNA methylation of the CpG islands of *Nlgn3*, *Setd1b*, and *Taok2* by pyrosequencing showed no significant differences between the study groups in adolescence (Figure 3D), while in adulthood, only a tendency toward decreased methylation levels of the *Taok2* promoter in females exposed to a maternal HFD was observed (*p* = 0.063) (Figure 3E).

### 2.4. Maternal HFD Alters the Levels of ASD-Related Proteins and the Expression of Mirnas in Adolescent Offspring PFCx

**Figure 4:**
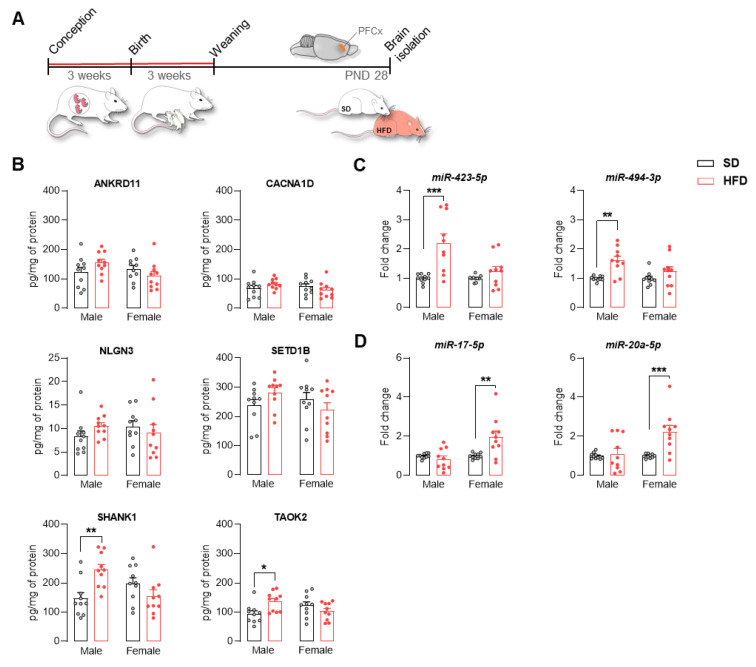
A maternal high-fat diet (HFD) during pregnancy and lactation increased SHANK1 and TAOK2 protein levels in the prefrontal cortex (PFCx) of adolescent male offspring. (**A**) Schematic of the protein analysis. Rat dams were fed a standard diet (SD) or HFD during pregnancy and lactation. After weaning, offspring were changed to an SD and sacrificed at postnatal day (PND) 28 to evaluate the changes in selected proteins and miRNA expression in the PFCx. (**B**) Effect of the maternal HFD on ANKRD11, CACNA1D, NLGN3, SETD1B, SHANK1, and TAOK2 protein levels in the PFCx of male and female offspring at PND 28. *SHANK1* (main effect of diet: F(1, 36) = 1.80, *p* = 0.19), diet × sex interaction: F(1, 36) = 12.40, *p* < 0.01); TAOK2 (main effect of diet: F(1, 36) = 1.09, *p* = 0.30), diet × sex interaction: F(1, 36) = 8.24, *p* < 0.01). (**C**,**D**) Effect of the maternal HFD on the miR-423 and miR-494 (**C**) and miR-17 and miR-20a (**D**) levels in the PFCx of male and female offspring at PND 28. miR-423 (main effect of diet: F(1, 36) = 15.78, *p* < 0.001), diet × sex interaction: F(1, 36) = 7.28, *p* < 0.05); miR-494 (main effect of diet: F(1, 36) = 13.75, *p* < 0.001), diet × sex interaction: F(1, 36) = 2.72, *p* = 0.11); miR-17 (main effect of diet: F(1, 36) = 4.61, *p* < 0.05), diet × sex interaction: F(1, 36) = 9.47, *p* < 0.01); miR-20a (main effect of diet: F(1, 36) = 9.39, *p* < 0.01), diet × sex interaction: F(1, 36) = 6.61, *p* < 0.05).Data are expressed as the mean ± SEM. *n* = 10 male and 10 female rats/experimental group. Data were analyzed by two-way ANOVA and the Bonferroni multiple comparison post hoc test. * *p* < 0.05, ** *p* < 0.01, *** *p* < 0.001 versus SD.

We assessed the effect of the maternal HFD diet on the level of selected proteins in the PFCx of offspring at PND 28 (Figure 4A). Among the analyzed proteins, namely, ANKRD11, CACNA1D, NLGN3, SETD1B, SHANK1, and TAOK2, a significant increase in the levels of SHANK1 (*p* < 0.01) and TAOK2 (*p* < 0.05) was observed in the group of males (Figure 4B). Additionally, we assessed the expression of selected miRNAs that can potentially interact with the selected proteins, including miR-423-5p and miR-494-3p (for SHANK1), as well as miR-17-5p and miR-20a-5p (for TAOK2). We observed increased expression of miR-423-5p and miR-494-3p in adolescent males exposed to a maternal HFD (*p* < 0.01) (Figure 4C). On the other hand, the expression of miR-17-5p and miR-20a-5p was increased in females from the HFD group (*p* < 0.01) (Figure 4D).

## 3. Discussion

To support that the development of ASD is caused by a complex interaction between genetic and environmental factors (e.g., diet), mainly related to epigenetic mechanisms [20], and because the pathogenesis of ASD is thought to mainly lie in the periods of the three trimesters of pregnancy and the initial postpartum period [21,22], we investigated the effect of exposure to modified maternal diets (HFD, HCD, and MD) limited to pregnancy and lactation on the susceptibility to ASD symptoms in male and female rat offspring.

First, within the PFCx of offspring, we assessed changes in the expression of 23 ASD-related genes. We noted changes in the expression of genes related to synaptic function, transcription processes, and chromatin remodeling, depending on the type of maternal diet, sex, and age. In adolescent male offspring, changes were induced by the maternal HFD (increased expression of *Cacna1d*, *Nlgn3*, *Shank1*, and *Taok2*), and in females, changes were mainly induced by the HCD (increased expression of *Itgb3*, *Slc6a4*, *En2*, and *Setd1b*). On the other hand, in early adulthood, the expression of *Mecp2* and *Setd1b* was increased in males from the HFD group, and the *Pten* and *Fmr1* mRNA levels in females from all groups (HFD, HCD, and MD) were decreased. Expression changes as well as mutations in genes whose mRNA levels were disturbed by the maternal diet were noted in patients with ASD in various brain structures that are important in the pathogenesis of this disorder [20,21,22,23,24,25,26]. It is worth emphasizing that ASD-associated genes, including genes whose proper expression was disturbed by the maternal diet, are characterized by functional pleiotropy and are crucial for proper brain development in the early stages and subsequent brain functioning. Moreover, the direction and time at which these pleiotropic pathways are dysregulated will lead to different, even opposing, effects, resulting in prenatal and postnatal neural and clinical heterogeneity reported in patients diagnosed with ASD [21]. Transcriptomic studies indicate that the different brain structures of subjects with ASD are characterized by the simultaneous down- and upregulation of numerous genes, and, for some genes, the opposite direction of changes is noted depending on the study (see review [27]). Furthermore, the profile of gene expression changes with age [28]. Because ASD is a childhood disease that occurs much more often in boys than in girls, in our further research, we decided to choose the maternal HFD because it most disturbed the expression of the studied genes at PND 28. Our previous research also showed that among the studied diets, the HFD led to the most modified transcriptome expression in the brain and behavior in offspring [29,30].

At the behavioral level, a maternal HFD during pregnancy and lactation led to autistic-like behaviors in offspring with marked changes mainly in adolescence. In the open field test, we observed significantly increased rearing episodes in adolescent male and female offspring and increased sector crossing and movement time only in adult males. These observations would indicate hyperactive and exploratory behavior or increased risk assessment and are supported by previous studies showing that maternal HFD during gestation caused hyperactivity in male offspring [31].

Next, a marble burying test was performed as a behavioral task reflecting repetitive and perseverative behavior [32]. Stereotypical marble burying behavior was significantly increased in adolescent male offspring from HFD dams. Evidence suggests that maternal HFD consumption 8 weeks prior to and during pregnancy and lactation also induces increased marble burying in offspring [6]. Increased repetitive behavior, that is, increased marble burying, was also observed in BTBR mice (a mouse line considered an idiopathic model of ASD) [33]. We also confirmed the increase in repetitive behavior in the offspring from the HFD group by assessing self-grooming, a parameter that is commonly used in the assessment of autistic-like behavior in rodents [34,35]. Males from the HFD group at PND 32 had increased self-grooming and decreased latency to self-grooming. Another study indicated that maternal obesity induced by 10 weeks of HFD feeding increased grooming in male but not female mouse offspring [36]. Increased self-grooming is also characteristic of preclinical ASD models, such as BTBR mice [34,37].

One of the core symptoms of ASD is impaired sociability and communication deficits [38,39]. Moreover, in individuals with ASD, aggression rates may be higher than those in healthy people [40,41]. We observed significantly reduced social interactions and increased fighting episodes during adolescence (this difference was also observed in adulthood) in male offspring rats exposed to a maternal HFD limited to pregnancy and lactation. Our results are consistent with other studies, which confirms that a maternal HFD and obesity cause social complications [6,42]. Moreover, mouse and rat offspring exposed to a maternal HFD are characterized by increased aggressive behavior in adulthood [43,44]. Furthermore, in another environmental ASD model, that is, prenatal exposure to valproic acid (VPA) [45,46], as well as in BTBR mice [7], decreased social episodes were noted. Altogether, our behavioral assessment indicates a strong relationship between a maternal HFD as a condition of predisposition to an autistic-like phenotype in adolescent male offspring, and our results correspond well with other preclinical autism models and epidemiological findings, showing a link between autism and obesity [39]. Interestingly, most of the behavioral disorders observed during adolescence disappeared in adulthood. One possible reason may be that the offspring were switched to a SD after weaning. Similar observations were made by Paradis and colleagues, who, after prenatal exposure to a diet rich in fat, noted a disturbance of fat preference in offspring in adolescence and its normalization in adulthood. At the same time, behavioral changes over the lifetime of the offspring correlated with specific brain molecular signatures [2]. It is worth emphasizing that long-term exposure to an HFD before and during pregnancy, inducing maternal obesity, resulted in significant brain alterations in mouse offspring that persisted into adulthood despite the offspring being switched to a low-fat diet at weaning [47]. Thus, the duration of exposure to an HFD and the presence of maternal obesity at the time of conception may play an important role in brain changes over the course of the offspring’s lifetime, which may be due to varying intrauterine conditions (e.g., the intensity of inflammation) that should be carefully investigated in subsequent studies.

On the other hand, the lack of an autistic-like phenotype in the female offspring of mothers consuming an HFD can be explained in two ways. First, research indicates the protective influence of female sex on the development of ASD symptoms, known as the female protective model: the need for a greater etiologic load for the phenotype to occur; the presence of a greater number of compensatory mechanisms; and molecular, structural, and hormonal differences compared to males. For example, the prevalence of putative functional de novo mutations (loss-of-function and predicted deleterious missense mutations) in female ASD patients is higher than that in males. In addition, prenatal testosterone exposure also plays an important role in the sex differences in ASD development. Second, it is worth emphasizing the sex-dependent pattern of clinical symptoms in patients with ASD in which males are mainly characterized by social deficits as well as repetitive and aggressive behavior, while females are characterized by a more frequent occurrence of low intellectual level and greater internalizing symptoms [48,49,50]. Hence, certain disorders related to the ASD phenotype in females from the HFD group could not be detected in behavioral tests focusing on the assessment of social and repetitive behaviors. A clear explanation of the mechanisms of the sex differences requires further detailed research; therefore, the use of males and females in research on ASD seems necessary.

DNA methylation in the PFCx of offspring after exposure to a maternal HFD was analyzed to understand the mechanism associated with the dysregulation of ASD-related gene expression. The multigenic condition of ASD seems to be dependent on gene–environment interactions. One of the potential responsible mechanisms, that is, epigenetic regulation, including DNA methylation, transcriptional regulation, and posttranslational regulation, is relevant to the neurodevelopmental processes that can be affected in utero by maternal lifestyle [51,52]. DNA methylation in CpG-rich promoters or gene regulatory regions is associated with inhibition of gene expression [53]. In postmortem studies of the brains of ASD patients, significant differences in DNA methylation (mainly cortical regions compared to healthy people) were noted [54]. In our findings, a maternal HFD enhances global DNA methylation in adolescent males, which may be due to increased DNMT3B protein levels (an enzyme associated with DNA methylation and the establishment of de novo methylation patterns) [55]. Switching to an SD after weaning restores global DNA methylation control levels in adulthood. Other studies have confirmed the impact of the maternal diet on disturbances in the level of DNA methylation in the brain and peripheral tissues of offspring throughout their lifetime [53,56,57]. In male but not female mice exposed to a maternal HFD, a decreased level of DNA methylation was observed in the PFCx at 6 weeks of age [56], while at 50 weeks of age, cortical DNA methylation levels were lowered in both females and males [57]. Hypermethylation of the *Pomc* promoter was observed in the hypothalamus at PND 22 in the offspring of HFD-treated dams [58]. Despite significant changes in global DNA methylation in male offspring, we found no changes in the methylation status of the promoters of the *Nlgn3*, *Setd1b*, and *Taok2* genes, which could explain their increased expression in our model. It is worth noting that the observation of increased DNA methylation at PND 28 correlates with our previous RNA-seq studies in which most (45) among the top 75 differentially expressed genes affected by maternal diet were downregulated in the HFD group at the same time point, in the frontal cortex [30]. Our data indicate that an altered *intrauterine* developmental environment is crucial for the DNA methylation pattern in the offspring’s brain (impaired in ASD individuals), but this epigenetic mechanism does not account for all the changes in gene expression, and other components of the epigenetic machinery should be investigated to fully explain the observed changes.

Among the investigated proteins, we noted a significant increase in the concentration of SHANK1 and TAOK2 in the PFCx of adolescent male offspring from the HFD group. Many studies have shown that mutations in *Shank* family genes lead to the development of ASD. Shank proteins are encoded by the three genes *Shank1*, *Shank2*, and *Shank3*, which are differentially expressed according to the region and stage of brain development and the cell type. Shank1 is a scaffolding protein that localizes to postsynaptic sites of excitatory synapses in the brain and is required for the proper formation and function of neuronal synapses. Moreover, SHANK1 is associated with scaffolds in the structural and functional organization of the dendritic spine and synaptic junction [26,59]. Interestingly, our research indicated increased expression of cortical markers of excitatory neurons in the offspring from the HFD group at PND 28 [30], which could explain the increased concentration of SHANK1 protein. Increased neuron numbers within the PFCx are noted in children with ASD [17]. Moreover, disturbed cortical excitation–inhibition balance is considered to be one of the pathophysiological mechanisms of ASD [60,61,62]. TAOK2 belongs to the MAP kinase kinase kinase (MAP3K) family, which regulates the p38 MAPK, SAPK/JNK and Hippo signaling pathways [63]. *Taok2* participates in the regulation of mitotic cell morphology, neuron development (dendrite formation and axon elongation) [64], and maturation of dendritic spines [65]. A recent study conducted by Richter and colleagues showed that mice lacking *Taok2* had abnormalities in brain size and neural connectivity, deficits in cortical layering, and abnormalities in the formation of dendrites and synapses [25]. Moreover, in *Taok2* and *Shank1* knockout mice, social and communication deficits were observed, respectively [25,66].

Among the miRNAs (short noncoding RNAs) that could potentially control the levels of SHANK1 (miR-423 and miR-494) and TAOK2 (miR-17 and miR-20a) posttranscriptionally, we noted an increase in the expression of miR-423 and miR-494 in adolescent males and miR-17 and miR-20a in females exposed to a maternal HFD. It is worth noting that increased expression of miR-494 in the PFCx was noted in a postmortem study of individuals diagnosed with ASD [67]. Although the observed increase in the expression of the miRNAs tested did not correlate with the changes in protein levels (miRNAs act mainly by degrading the mRNA transcripts or repressing translation [68]), it is worth emphasizing that a single mRNA transcript can be a target for many miRNAs, and a given miRNA is capable of interacting with numerous mRNA transcripts [69]. On the other hand, increased expression of miR-423 and miR-494 may be an adaptive mechanism whose function is to restore the normal level of SHANK1. Thus, the observed changes in expression may indicate a disturbance in the machinery controlling translation, are consistent with the growing body of data highlighting the role of miRNAs in the pathogenesis of ASD [68,69], and encourage a deeper analysis of the mechanisms of the maternal diet effects on the development of offspring. Evidence confirms that maternal nutrition could modify miRNA expression profiling in offspring through lipid metabolism, insulin resistance, and inflammation [70].

In the context of behavioral disturbances associated with ASD, it is worth noting the existence of other important peripheral and central disorders in the offspring exposed to a maternal HFD that were not assessed in this study. Clinical studies report that individuals with neurodevelopmental disorders, including ASD, have coexisting gastrointestinal problems and dysbiosis of the gut microbiota [71]. The gut–brain axis is a complex bidirectional communication network mediated by hormonal, immune, and nervous signals between the gut and the brain [72]. Recent findings support the relationship between behavioral dysfunction in offspring associated with a maternal HFD and changes in the gut microbiota of the offspring [6,73,74]. One of the potential mechanisms of behavioral dysfunction could be associated with an HFD through alterations in the offspring gut microbiota. Moreover, treatment with a single species of commensal bacteria has been shown to correct oxytocin levels and synaptic dysfunction and to reverse social deficits in offspring [6]. In addition, both probiotics and oxytocin have been reported to have therapeutic potential in the treatment of ASD [75]. A maternal HFD may also interfere with the proper functioning of the immune system in the brains of offspring, but in a previous study by our group, we did not notice changes in the levels of proinflammatory cytokines (interleukin-1α, interleukin-6, and tumor necrosis factor-α) in the hippocampi of offspring at PND 28 [29]. An additional limitation of this study is that we analyzed molecular changes only within the PFCx, while other brain regions, such as the amygdala, hippocampus, or cerebellum, are also involved in the pathogenesis of ASD [76,77].

Taken together, our research highlights the important role of a maternal HFD limited to pregnancy and lactation in the risk of developing an autistic-like phenotype in male offspring. Thus, we confirm the key role of the prenatal and early postnatal development environment modulated by the maternal diet in the proper development of the brain or the predisposition of neurodevelopmental diseases. Importantly, returning to a fully balanced diet after lactation may reduce some of the negative changes in the offspring. On the other hand, the heterogeneity of the changes in the brain depending on the type of maternal diet, development period, and sex of the offspring, in a similar way to the situation regarding the great heterogeneity in the pathogenesis and the symptoms of ASD, prompts further studies of the relationship between the maternal diet and the development of ASD.

## 4. Materials and Methods

### 4.1. Animals and Diets

All procedures were performed following EU Directive 2010/63/EU with the approval of the Ethical Committee at the Maj Institute of Pharmacology Polish Academy of Sciences (approval numbers 1270/2015, 17 December 2015, and 18/2020, 23 January 2020).

Animals were maintained on a 12 h/12 h light/dark cycle (lights on at 6:00 a.m.) at 22 ± 2 °C and 55 ± 10% humidity with food and water ad libitum. Virgin female Wistar rats (*n* = 30, bodyweight: 200–240 g) were purchased directly from Charles River (Sulzfeld, Germany). After acclimatization and during the proestrus phase (smears from females were assessed to determine the estrous cycle phase), males were mated with the females overnight. After confirmation of pregnancy by determining the presence of sperm in the vaginal smear, rats were housed individually and randomly assigned to one of four groups. The control group of dams (*n* = 9) was fed a standard diet (SD; 65% carbohydrate, 13% fat, 22% protein, 3.4 kcal/g; VRF1; Special Diets Services, Witham, UK). Other groups of dams were fed one of three modified diets during the pregnancy and lactation periods that were purchased from Altromin (Lage, Germany): high-fat diet–(HFD; 24% carbohydrate, 60% fat, 16% protein, 5.31 kcal/g; C1057 mod.; *n* = 9); high-carbohydrate diet–(HCD; 70% carbohydrate: rich in sucrose–40%, 12% fat, 18% protein, 3.77 kcal/g; C1010; *n* = 6); or mixed diet–(MD; 56% carbohydrate, 28% fat, 16% protein, 3.90 kcal/g; C1011; *n* = 6). The modified maternal diets used in this study did not affect the litter size or birth weight of offspring. Moreover, HFD-, MD-, and HCD-fed dams during pregnancy and lactation did not have significant differences in body weight compared to dams from the SD group (except during the last week of lactation, when the body weight of MD dams was lower) [78].

On day 1 after birth, litter sizes were normalized to 10–12 pups with a sex ratio as close to 1:1 as possible. At weaning postnatal day (PND) 22, offspring were separated according to sex and housed 5 per cage, and the offspring were kept on standard chow until the end of the study. In each of the behavioral and molecular experiments, 10 male and 10 female offspring from each experimental group (SD, HFD, HCD, and MD) were used. To reduce “litter effects”, animals for each group were selected from 3 to 4 different dams [79].

All behavioral experiments were performed during the light phase of the light/dark cycle (9:00 a.m. to 01:00 p.m.). Offspring rats were adapted to the experimental room 1 h prior to behavioral tasks.

### 4.2. Open Field Test

The procedure was performed as described previously [80], with some modifications. An open field test was performed at PND 28 and PND 63 in offspring from the SD and HFD groups. The testing arena (black open field box, 60 × 60 cm with 35 cm walls) was divided into 16 squares with white lines (15 × 15 cm). The four squares located in the center of the box were defined as the central zone, and the others (12) were defined as the peripheral arena. The experimental room was dark, and only the center of the open field was illuminated by a 75 W incandescent lamp placed 70 cm above the apparatus floor. At the start of the test, the animals were placed gently in the center of the platform and allowed 5 min of spontaneous activity. Their exploratory activity in the open field, i.e., walking time, number of sector line crossings (ambulations), time spent in the center zone, number of entries to the center, and episodes of rearing, was measured. The testing arena was cleaned with 70% alcohol and air-dried between each trial for every rat.

### 4.3. Marble Burying Task

The procedure was performed at PND 30 and PND 65 in offspring from the SD and HFD groups. Each rat was placed in a clear polycarbonate cage (23 × 39 × 18 cm) with sawdust bedding (5 cm thick). Twenty glass marbles (1.5 cm in diameter) were placed in five rows, with each row containing four marbles. The total number of marbles that were covered (two-thirds were buried) was counted after 30 min. Marbles and cages were cleaned with 70% ethanol and air-dried, and the cage was refilled with new bedding materials between every individual test.

### 4.4. Self-Grooming

The procedure was performed as described previously [34] at PND 32 and PND 67 in offspring from the SD and HFD groups. Rats were gently placed in clear polycarbonate cages (23 × 39 × 18 cm) without bedding and allowed to freely explore the cage for the entirety of the test. The total recording time lasted 20 min. The first 10 min was considered a habituation period, and the second 10 min was considered a testing period when the cumulative time spent grooming (stroking, scratching, or licking head or body parts) was measured. The cage was cleaned with 70% alcohol and air-dried between each trial for every rat.

### 4.5. Social Interaction Test

The procedure was performed as previously described [6], with some modifications, at PND 34 and PND 69 in offspring from the SD and HFD groups. The social interaction test was conducted using a black box (60 × 60 × 35 cm). Two unfamiliar (selected from separate housing cages) rats of the same sex, the same experimental group, and matched for body weight (±5 g for adolescent rats and ±10 g for adult rats) were diagonally placed in opposite corners of the box facing away from each other. The social interaction of the animals was measured over a 10 min period. The test box was wiped clean with 70% ethanol and air-dried between each session. Social interaction between two rats was expressed as the total time spent in social behavior, such as sniffing, genital investigation, chasing, and fighting with each other. Moreover, the number of contacts and episodes of sniffing, fighting, and close following were counted. Each rat was tested twice with another partner for a total of 10 pairs. Data from the social interaction test were expressed as a summed score for each pair of rats.

### 4.6. Brain Tissue Collection

The subsets of offspring from the SD, HCD, HFD, and MD groups were sacrificed through rapid decapitation at PND 28 and PND 63. The PFCx (including the infralimbic, prelimbic, and cingulate cortices; bregma: 5.2–2.7 mm) was dissected according to The Rat Brain Atlas [81]. The structures were immediately frozen on dry ice and stored at −80 °C until further molecular analysis. To avoid the potential effect of stress on molecular changes in the brain, animals were not fasted before decapitation. All samples were collected between 9:00 a.m. and 12:00 p.m.

### 4.7. Taqman Gene Expression Array Cards

RNA was isolated following the manufacturer’s protocol using the RNA Mini Kit (A&A Biotechnology, Gdańsk, Poland). The total RNA concentration was measured using an ND-1000 Spectrometer (NanoDrop Technologies Inc., Wilmington, DE, USA). One microgram of total RNA was reverse transcribed into cDNA using a High-Capacity cDNA Reverse Transcription Kit (Applied Biosystems, Waltham, MA, USA).

In this study, we used 384-well TaqMan Gene Expression Custom Array Cards 24 (Cat# 4342249, Applied Biosystems), designed to cover different gene families relevant to ASD, selected based on the literature, the SFARI database, and previous RNA-seq analyses from the frontal cortex in the same model (PRJNA669556 BioProject) [30]. The gene set listed in Supplementary Table S1 and housekeeping genes (18S rRNA and Hprt1) were studied. Real-time (RT)-qPCR was carried out using Life Technologies TaqMan reagents (e.g., TaqMan Fast Advanced Master Mix) according to the manufacturer’s protocol using a QuantStudio 12K Flex Real-Time PCR System (Applied Biosystems). Data were further analyzed with QuantStudio 12K Flex Expression Suite Software (Applied Biosystems). Quantification cycle data were normalized to 18S rRNA. The relative gene expression was calculated using the 2^−∆∆Ct^ (fold change) method.

### 4.8. Analysis of Gene Expression by RT-qPCR

Reverse transcription to cDNA was performed using a High-Capacity cDNA Reverse Transcription Kit (Applied Biosystems). RT-qPCR was performed in duplicate on a 96-well plate using QuantStudio 3 (Applied Biosystems). Gene expression was assessed with the use of the following TaqMan Gene Expression Assays (Thermo Fisher Scientific): *Taok2* (assay ID: 00666184), Setd1b (assay ID: 01507821), Dnmt1 (assay ID: 00709664), Dnmt3a (assay ID: 01027162), and Dnmt3b (assay ID: 01536419). The following conditions were used: an initial step of 95 °C for 10 min, followed by 40 cycles of 95 °C for 15 s and then 60 °C for 60 s. The 18S rRNA (assay ID: 99999901) and Hprt1 (assay ID: 01527840) expression levels were used as housekeeping controls, and the values are expressed as the fold change from the control (SD group).

### 4.9. Analysis of miRNA Expression by RT-qPCR

Total RNA (20 ng) and miRNA-specific stem-loop RT primers (Applied Biosystems) were used for the reverse transcription reactions of miRNA. The cDNAs were then synthesized with the TaqMan MicroRNA Reverse Transcription Kit (Applied Biosystems) according to the manufacturer’s protocol. RT-qPCR was performed with TaqMan MicroRNA assays (Applied Biosystems) to analyze the expression of the following mature miRNAs: miR-423 (assay ID: 465126), miR-494 (assay ID: 462468), miR-20a (assay ID: 000580), and miR-17 (assay ID: 002308), selected based on the highest target and rank scores in the miRDB database for miRNA target prediction and functional annotations (http://mirdb.org, accessed date: 16 December 2020). The relative amount of each miRNA was assessed using the comparative CT method (2^−ΔΔCt^) and normalized to the U6 small nuclear RNA (U6 snRNA).

### 4.10. Enzyme-Linked Immunosorbent Assay (ELISA)

In adolescent offspring, we assessed the level of proteins encoded by selected genes with altered expression due to the maternal diet. The protein levels of ANKRD11 (Cat# E2515Ra), CACNA1D (Cat# E2558Ra), NLGN3 (Cat# E2338Ra), SETD1B (Cat# E2517Ra), SHANK1 (Cat# E2516Ra), TAOK2 (Cat# E2606Ra), and DNMT3B (Cat# E2657Ra) were measured using ELISA kits (Bioassay Technology Laboratory, Shanghai, China) in accordance with the manufacturer’s protocol. Duplicates of each sample and series of standards were transferred to ELISA plates. The absorbance was measured at a wavelength of λ = 450 nm using a Multiskan Spectrum spectrophotometer (Thermo LabSystems, Philadelphia, PA, USA). The concentration of proteins was calculated from a standard curve and expressed as pg/mg of protein. For protein measurement, the Pierce BCA Protein Assay Kit (Thermo Scientific, Rockford, IL, USA) was used. 

### 4.11. Quantify Global DNA Methylation

DNA was isolated following the manufacturer’s protocol using the AllPrep DNA/RNA/Protein Mini Kit (Qiagen, Hilden, Germany). One hundred nanograms of DNA were used for the study. The level of global DNA methylation (5-mC%) was measured using a MethylFlash Global DNA Methylation (5-mC) ELISA Easy Kit (Cat# P-1030-96; Epigentek, Farmingdale, NY, USA) according to the manufacturer’s instructions. The absolute quantification of 5-mC% contents was performed using a standard curve according to the manufacturer’s manual.

### 4.12. Assessment of DNA Methylation Level of the CpG Islands by Pyrosequencing

To determine whether DNA methylation is one of the mechanisms responsible for the regulation of the expression of selected genes, bisulfite pyrosequencing tests were performed. DNA was isolated from the PFCx using the AllPrep DNA/RNA/Protein Mini Kit (Qiagen). The study group consisted of 80 DNA samples divided into 8 subgroups depending on sex (male/female), mother’s diet (SD/HFD), and days of life (28/63). Five hundred nanograms of DNA was bisulfite treated using an EpiTect Plus bisulfite kit (Qiagen) according to the manufacturer’s protocol. Target regions were designed to amplify fragments of CpG islands localized approximately up to 1500 bp upstream of the gene or within the first exon of selected genes. The exact localization of the tested CG nucleotides is listed in Table S2. Primers for both amplicon preparation and sequencing for each selected gene were designed with PyroMark Assay Design Software 2.0.1.15 (Qiagen). One of the primers used for amplicon preparation was biotin labeled at the 5′ end. Detailed information about the primer sequences, genomic localization (RGSC 6.0/rn6), amplicon sizes, and annealing temperatures is listed in Table S3. PCR was carried out with the use of a PyroMark PCR kit (Qiagen). The composition of the PCR mixture was as follows: 1 µL of converted DNA, 1× PyroMark Master Mix (HotStarTaq DNA Polymerase, dNTPs, PyroMark PCR Buffer), 10 pmol of each primer, and 1× Coral Load Concentrate. PCR specificity was checked by electrophoresis in a 1.8% agarose gel stained with SimplySafe (EURx, Gdańsk, Poland) and visualized under UV light. Pyrosequencing assays for each gene were designed with the use of PyroMarkQ48 Autoprep 2.4.2 Software (Qiagen). Each assay was designed to include a conversion control. Pyrosequencing analysis additionally included fully methylated and unmethylated controls. A fully methylated control was prepared by incubating 500 ng of DNA with 1 µL of CpG methyltransferase (Thermo Scientific™, Carlsbad, CA, USA), 1× reaction buffer (Thermo Scientific™), and 1× S-adenosylmethionine (Thermo Scientific™) as a cofactor at 37 °C for 1 h. One hour later, 1 µL of CpG methyltransferase was added and incubated for another 1 h at 37 °C. The reaction was stopped by increasing the temperature to 65 °C for 20 min. The product of whole genome amplification (GenomePlex^®^ Complete Whole Genome Amplification (WGA) Kit, (Sigma-Aldrich, St. Louis, MO, USA)) was used as a fully unmethylated control. The pyrosequencing test was performed according to the manufacturer’s protocol with the use of PyroMark Q48 Advanced CpG Reagents (Qiagen) and PyroMark Q48 Autoprep (Qiagen). As a result, we obtained the average percentage methylation value in each sample.

### 4.13. Statistical Analyses

All data are expressed as the mean ± standard error of the mean (SEM). Data were analyzed with two-way ANOVA (diet × sex) followed by a Bonferroni post hoc test using GraphPad Prism 9.1.0 software (GraphPad Software, La Jolla, CA, USA). A value of *p* < 0.05 was considered statistically significant.

## Figures and Tables

**Table 1:** Results of statistical analysis for genes: which expression was altered in the offspring PFCX after exposure to maternal modified diets during pregnancy and lactation.

Gene	Main Effect of Diet	Diet × Sex Interaction
PND 28		
*Ankrd11*	F(3, 72) = 3.06, *p* < 0.05	F(3, 72) = 0.45, *p* = 0.72
*Cacna1d*	F(3, 72) = 1.28, *p* = 0.29	F(3, 72) = 3.17, *p* < 0.05
*En2*	F(3, 72) = 4.67, *p* < 0.01	F(3, 72) = 0.10, *p* = 0.96
*Itgb3*	F(3, 72) = 4.29, *p* < 0.01	F(3, 72) = 2.84, *p* < 0.05
*Nlgn3*	F(3, 72) = 0.96, *p* = 0.42	F(3, 72) = 3.03, *p* < 0.05
*Shank1*	F(3, 72) = 0.92, *p* = 0.44	F(3, 72) = 3.46, *p* < 0.05
*Slc6a4*	F(3, 72) = 2.96, *p* < 0.05	F(3, 72) = 1.33, *p* = 0.27
*Setd1b*	F(3, 72) = 5.10, *p* < 0.01	F(3, 72) = 24.44, *p* < 0.001
*Taok2*	F(3, 72) = 4.71, *p* < 0.01	F(3, 72) = 0.68, *p* = 0.57
PND 63		
*Fmr1*	F(3, 72) = 8.23, *p* < 0.001	F(3, 72) = 2.80, *p* < 0.05
*Itgb3*	F(3, 72) = 5.07, *p* < 0.01	F(3, 72) = 5.04, *p* < 0.01
*Mecp2*	F(3, 72) = 5.41, *p* < 0.01	F(3, 72) = 2.51, *p* = 0.07
*Pten*	F(3, 72) = 2.55, *p* = 0.06	F(3, 72) = 3.02, *p* < 0.05
*Reln*	F(3, 72) = 5.06, *p* < 0.01	F(3, 72) = 0.29, *p* = 0.83
*Shank2*	F(3, 72) = 4.32, *p* < 0.01	F(3, 72) = 0.24, *p* = 0.86
*Setd1b*	F(3, 72) = 25.82, *p* < 0.001	F(3, 72) = 7.81, *p* < 0.001
*Taok2*	F(3, 72) = 0.87, *p* = 0.97	F(3, 72) = 9.89, *p* < 0.001

## Data Availability

All relevant data is presented in the manuscript, raw data is available upon request from the corresponding author.

## Supplementary Materials

The following are available online at https://www.mdpi.com/article/10.3390/ijms22189662/s1.
