# Supplementary material for: Alteration of the Early Development Environment by Maternal Diet and the Occurrence of Autistic-like Phenotypes in Rat Offspring
Source: Int J Mol Sci. 2021 Sep 7;22(18):9662. doi: 10.3390/ijms22189662 (PMC8472469; doi:10.3390/ijms22189662)
Supplement: Supplementary file 1 [file ijms-22-09662-s001.zip › ijms-1320924 Table supplementary.pdf]

Table S1. TaqMan Gene Expression Array gene list.

Gene Symbol	Gene Name	Reference Life Technologies
Ankrd11	Ankyrin Repeat Domain 11	Rn01431699_m1
Bcl11a	B-Cell CLL/Lymphoma 11A (Zinc Finger Protein)	Rn01225236_m1
Cacna1d	Calcium Channel, Voltage-Dependent, L Type, Alpha 1D Subunit	Rn01453395_m1
Cacna2d3	Calcium Channel, Voltage-Dependent, Alpha2/Delta Subunit 3	Rn00598241_m1
En2	Engrailed Homeobox 2	Rn01410380_m1
Fmr1	Fragile X Mental Retardation 1	Rn00709627_m1
Foxp2	Forkhead Box P2	Rn01456150_m1
Gabrb3	Gamma-Aminobutyric Acid (GABA) A Receptor, Beta 3	Rn00567029_m1
Hprt1	Hypoxanthine Phosphoribosyltransferase 1	Rn01527840_m1
Htr2a	5-Hydroxytryptamine (Serotonin) Receptor 2A	Rn00568473_m1
Itgb3	Integrin, Beta 3	Rn00596601_m1
Mecp2	Methyl Cpg Binding Protein 2	Rn01529606_g1
Nlgn3	Neuroligin 3	Rn01419002_m1
Oxtr	Oxytocin Receptor	Rn00563503_m1
Pten	Phosphatase and Tensin Homolog	Rn00477208_m1
Reln	Reelin	Rn00589609_m1
Shank1	SH3 and Multiple Ankyrin Repeat Domains 1	Rn00582088_m1
Shank2	SH3 and Multiple Ankyrin Repeat Domains 2	Rn01479040_m1
Shank3	SH3 and Multiple Ankyrin Repeat Domains 3	Rn00572344_m1
Slc6a4	Solute Carrier Family 6 (Neurotransmitter Transporter), Member 4	Rn00564737_m1
Syn1	Synapsin I	Rn00569468_m1
Wnt2	Wingless-Type MMTV Integration Site Family Member 2	Rn01500736_m1
Taok2	TAO Kinase 2	Rn00666184_m1
18S	Eukaryotic 18S rRNA	Hs99999901_s1

Table S2. The exact localization of the tested CG nucleotides during pyrosequencing experiments.

Region	Position	Chromosomal localization (RGSC 6.0/rn6)	
**Nlgn3**	Pos.1	chrX:71,199,517-71,199,518	Exon 1
	Pos.2	chrX:71,199,531-71,199,532	
	Pos.3	chrX:71,199,538-71,199,539	
	Pos.4	chrX:71,199,544-71,199,545	
	Pos.5	chrX:71,199,564-71,199,565	
	Pos.6	chrX:71,199,578-71,199,579	
	Pos.7	chrX:71,199,585-71,199,586	
	Pos.8	chrX:71,199,594-71,199,595	
**Taok2**	Pos.1	chr1:198,320,947-198,320,948	CpG island
	Pos.2	chr1:198,320,953-198,320,954	
	Pos.3	chr1:198,320,957-198,320,958	
	Pos.4	chr1:198,320,962-198,320,963	
	Pos.5	chr1:198,320,968-198,320,969	
	Pos.6	chr1:198,320,970-198,320,971	
	Pos.7	chr1:198,320,975-198,320,976	
	Pos.8	chr1:198,321,002-198,321,003	
	Pos.9	chr1:198,321,013-198,321,014	
**Setd1b**	Pos.1	chr1:199,224,075-199,224,076	CpG island
	Pos.2	chr1:199,224,085-199,224,086	
	Pos.3	chr1:199,224,098-199,224,099	
	Pos.4	chr1:199,224,101-199,224,102	
	Pos.5	chr1:199,224,110-199,224,111	
	Pos.6	chr1:199,224,130-199,224,131	
	Pos.7	chr1:199,224,149-199,224,150	
	Pos.8	chr1:199,224,164-199,224,165	
	Pos.9	chr1:199,224,171-199,224,172	

Table S3. Detailed information about primers sequences, genomic localization (RGSC 6.0/rn6), amplicon sizes and annealing temperatures during pyrosequencing experiments.

Region	Localization (RGSC 6.0/rn6)	Primer	Primer Sequence 5'-3'	Annealing Temperature	Product Size
Nlgn3	chrX:71,199,393-71,199,624	F	GGGTTATGTTTTAGGATTTGAGTTA	55 °C	232bp
		R	biotin-ACCTCACCTATTCCCTAAAC		
		S	GGTATAGTAGTTAGGTTG	Sequencig primer	
Taok2	chr1:198,320,924-198,321,070	F	AGGATTGTTGGAGAGAGTTGTA	61 °C	147bp
		R	biotin-TACTCCTTTCAACCTTTACCTTACA		
		S	TGTTGGAGAGAGTTGTA	Sequencig primer	
Setd1b	chr1:199,224,035-199,224,203	F	GGTTTTTTGAGTTGTTTTATTAAGAA	55 °C	169bp
		R	biotin-CCCAAATACCTATAAAACTACAATACA		
		S	GTTTTATTAAGAAAAATTTAGGAATAG	Sequencig primer	

F – Forward primer; R – Reverse primer

	Step	Temp [°C]	Time	
**Pyrosequencing PCR**	Initial heat activation	95	15 min	
	Denaturation	94	30 s	45 cycles
	Annealing	55-61*	30 s	
	Extension	72	30 s	
	Final extension	72	10 min	

* Annealing temperature depends on the using primers
